# Maternal High-Fat Diet and Offspring Hypertension

**DOI:** 10.3390/ijms23158179

**Published:** 2022-07-25

**Authors:** You-Lin Tain, Chien-Ning Hsu

**Affiliations:** 1 Department of Pediatrics, Kaohsiung Chang Gung Memorial Hospital, Kaohsiung 833, Taiwan; tainyl@cgmh.org.tw; 2 College of Medicine, Chang Gung University, Taoyuan 333, Taiwan; 3 Department of Pharmacy, Kaohsiung Chang Gung Memorial Hospital, Kaohsiung 833, Taiwan; 4 School of Pharmacy, Kaohsiung Medical University, Kaohsiung 807, Taiwan

**Keywords:** hypertension, high-fat diet, developmental origins of health and disease (DOHaD), oxidative stress, nitric oxide, renin-angiotensin system, epigenetic regulation, gut microbiota

## Abstract

The incidence of hypertension has increased to epidemic levels in the past decades. Increasing evidence reveals that maternal dietary habits play a crucial role in the development of hypertension in adult offspring. In humans, increased fat consumption has been considered responsible for obesity and associated diseases. Maternal diets rich in saturated fats have been widely employed in animal models to study various adverse offspring outcomes. In this review, we discussed current evidence linking maternal high-fat diet to offspring hypertension. We also provided an in-depth overview of the potential mechanisms underlying hypertension of developmental origins that are programmed by maternal high-fat intake from animal studies. Furthermore, this review also presented an overview of how reprogramming interventions can prevent maternal high-fat-diet-induced hypertension in adult offspring. Overall, recent advances in understanding mechanisms behind programming and reprogramming of maternal high-fat diet on hypertension of developmental origins might provide the answers to curtail this epidemic. Still, more research is needed to translate research findings into practice.

## 1. Introduction

The growing occurrence of cardiometabolic disease is a worldwide health problem that influences all age groups, including women of childbearing age [1,2,3]. Today, a broad spectrum of factors contributes to cardiometabolic disease [3], including genetic susceptibility, intra-uterine growth retardation, imbalanced diet, socioeconomic status, physical activity, etc. Among them, imbalanced diet is a key modifiable factor that can be targeted to control cardiometabolic disease [4]. Recently, the increased consumption of fats, especially saturated fats, has raised interest in finding how high-fat diet increases susceptibility to cardiometabolic disease. A high-fat diet (HFD) is more reflective of the dietary habits in western society. Dietary patterns with high-fat consumption established in early childhood tend to continue during pregnancy.

A mounting body of epidemiological and experimental evidence supports a role for early-life environmental exposures in determining the long-term health of an individual, which is referred as the Developmental Origins of Health and Disease (DOHaD) theory [5]. Fetal development is dependent on maternal nutrition. Under- or over-nutrition has adverse effect on offspring [6]. A maternal HFD can alter the morphology and function of various tissues/organs and thus the offspring become more susceptible to many diseases later in life [7,8].

It is challenging to study maternal dietary patterns in human participants due to difficulty in exactly calculating nutrient content of food intake, and ethical issues related to manipulating the dietary patterns of pregnant women. Animal models provide an invaluable tool to control over diet composition, eliminate confounding factors, and explore the underlying mechanisms of developmental programming [9]. Animal models of maternal HFD demonstrate that adverse outcomes may occur in the offspring, such as obesity, insulin resistance, behavior disorders, endothelial dysfunction, hypertension, hepatic steatosis, dyslipidemia, increased visceral fat mass, glucose intolerance, and adipocyte hypertrophy, etc. [7,8,9,10,11].

Hypertension has a central role in cardiometabolic disease [3,4]. Although blood pressure (BP) shows multifactorial inheritance patterns, known genetic variants only explain ~3.5% of BP trait variability [12]. Increasing evidence indicates that developmental programming can take place in early life, resulting in hypertension later in life [13,14,15]. A wide array of early-life environmental stimuli can induce developmental programming of hypertension [13,14,15,16]. Among them, maternal nutrition has a role in the pathogenesis of hypertension of developmental origins [15]. Maternal HFD is considered as a key early-life trait involving adverse offspring outcomes. Nevertheless, little is known on whether maternal HFD can induce offspring hypertension and the underlying mechanisms.

To examine and identify the evidence around the impact of maternal HFD on offspring hypertension, our search strategy was developed for literature retrieval in the PubMed/MEDLINE databases on maternal HFD, DOHaD, and hypertension. We used the following search terms: “developmental programming”, “DOHaD”, “reprogramming”, “blood pressure”, “mother”, “pregnancy”, “gestation”, “lactation”, “offspring”, “progeny”, “high-fat diet”, and “hypertension”. We further checked fitting reference lists to find additional studies in eligible papers. The last search was conducted on 20 June 2022.

## 2. Maternal High-Fat Diet Programs Adult Diseases

Relatively little information currently exists concerning the impact of maternal HFD on offspring health in humans. Although some evidence links maternal obesity during pregnancy with an increased risk of obesity in offspring in later life [17,18], maternal obesity does not necessarily correspond to maternal high-fat diet [10]. Most epidemiological studies that pool various participants as well as fat components from different dietary sources bring great risk for diluting any real findings. In contrast, dietary composition can be easily determined for the purpose of comparisons in animal models across species. Hence, much of what is known about the implication of maternal HFD on offspring health is mainly based on animal models. Animal models have been instrumental in indicating the biological plausibility of the relations noticed in epidemiological research and provide proof of causality.

High-fat diets have long been known for generating obesity in animal models and associated diseases [18,19]. Nevertheless, this dietary intervention is not standardized, and the HFD-induced phenotypes vary distinctly among different studies [18,19]. Prior reviews demonstrated that different HF diets with fat fractions ranged from 20% to 60% energy as fat, and the basic constituents of fats vary between plant oils (e.g., corn or coconut oil) and animal-derived fats (e.g., lard or butter) [7,8,19]. Notably, diets rich in saturated fats can lead to health risks, while unsaturated fats are beneficial to heart health [20]. These obviously lead to a considerable variability in the results being reported.

The effect of a maternal HFD on the offspring has been examined in rats [7,8], mice [7,8], rabbits [21], pigs [22], and non-human primates [23]. A systematic review recruiting 17 animal studies demonstrated that maternal HFD may compromise parameters in feeding behavior and body composition of offspring [24]. Another systemic review including 11 studies identified the risk of type 2 diabetes and obesity in male offspring exposed to a maternal HFD [25].

**Figure 1:**
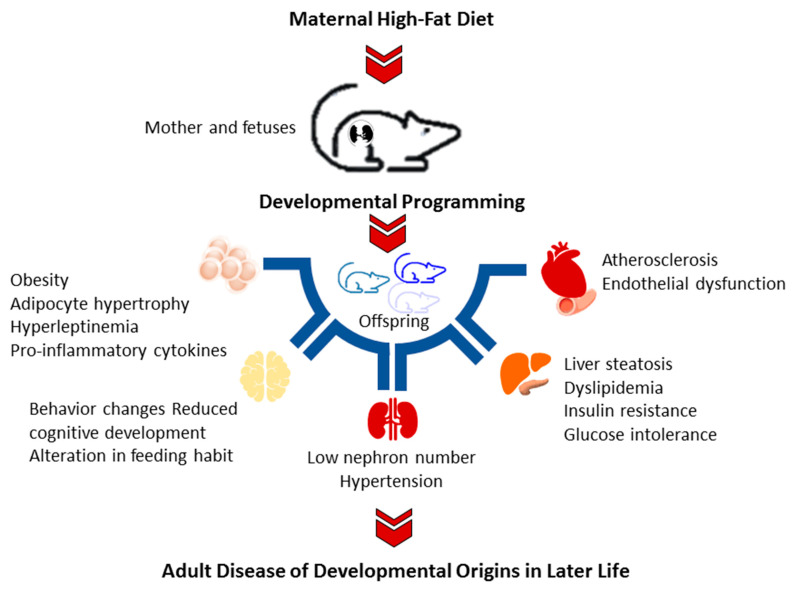
Schematic diagram summarizing the adverse offspring outcomes related to maternal high-fat diet.

Current evidence has emerged from animal models that offspring exposed to a maternal HFD manifest various components of metabolic syndrome [26,27], including obesity [26], insulin resistance [28], liver steatosis [29], dyslipidemia [29], and hypertension [30]. Additionally, maternal HFD may modify the development of the brain, resulting in reduced cognitive development, increased depressive-like and aggressive behaviors, and alteration in feeding habits in the offspring [8]. A brief summary of adverse offspring outcomes consequent of maternal HFD is depicted in Figure 1.

Although a variety of adverse offspring outcomes related to maternal HFD have been reported, at present, our understanding of how maternal HFD induces offspring hypertension, the mechanisms behind developmental programming, and efficient reprogramming strategies remain largely unknown.

## 3. High-Fat Diet and Hypertension

Regulation of blood pressure (BP) is a complex integrated response involving a variety of organ systems, including the heart, blood vessels, brain, and kidneys [28]. Besides, the maintenance of normal BP needs the interconnection and coordination of several regulatory mechanisms involving the nitric oxide (NO), renin–angiotensin system (RAS), the sympathetic nervous system, and sodium excretion [31].

### 3.1. Cardiovascular System

Observational studies in humans linked high saturated fat consumption with atherosclerosis and coronary artery disease, while monounsaturated fat consumption is association with reduction of cardiovascular mortality [32]. Endothelial cells are important constituents of blood vessels that determine cardiovascular homeostasis [33]. Dysfunction of endothelium has been characterized by a shift from executing physiologic functions of endothelium to a pro-inflammatory setting, a pro-thrombotic state, and vasoconstriction. All these together implicate in the pathogenesis of hypertension. Notably, these events can be induced by oxidative stress in the presence of high-fat intake [34]. The vasculature is a major source of NADPH oxidase-derived ROS [35]. High-fat diet causes endothelial dysfunction and vascular oxidative stress is related to increases of NADPH oxidase-derived ROS [36]. Additionally, several endothelium-derived vasoconstrictors, such as angiotensin II (Ang II) [37], urotensin II [38], and vasoconstrictor prostaglandins [39], can be released by endothelium in response to a high-fat diet.

On the other hand, reduced bioavailability of NO, a well-known vasodilator, is considered a hallmark of endothelial dysfunction [40]. The endothelial NO synthase (NOS)-derived NO is responsible for vasodilation in the cardiovascular system. Prior work indicated that high-fat-diet-induced endothelial dysfunction accompanying by reduced eNOS-derived NO [36]. High-fat diet can also increase asymmetric dimethylarginine (ADMA), an endogenous NOS inhibitor [41]. ADMA can uncouple NOS isoenzymes to form superoxide, contributing to endothelial dysfunction [42]. Together, high-fat diet disturbs vascular tone via regulating vasodilators and vasoconstrictors and causes vascular dysfunction, arterial stiffness, atherosclerosis, and vascular remodeling as well [43], all of which contribute to the development of hypertension.

### 3.2. Central Nervous System

The central nervous system (CNS) organizes regional sympathetic outflow to target organs (e.g., the kidneys and heart) through the integration of autonomic brainstem networks, reflex influences, and input from circulating factors [44]. Overexcitation of the sympathetic nervous system has a crucial role in the pathogenesis of hypertension [44]. Similar to the cardiovascular system, oxidative stress in the CNS is involved in the development of hypertension [45]. ROS increase sympathoexcitatory inputs to rostral ventrolateral medulla (RVLM) neurons, while iNOS-mediated NO production stimulates sympathoinhibition [46]. As a result, an imbalance of ROS and NO in the RVLM increases sympathetic tone, resulting in hypertension [45,46]. Conversely, interventions that reduce brain oxidative stress have been reported to prevent neurogenic hypertension [47].

Although mounting evidence supports that HFD promotes oxidative stress in the brain [48], so far only few reports demonstrated the impact of HFD on hypertension induced by brain oxidative stress [49,50].

### 3.3. Renal System

Several lines of evidence clearly indicate that the kidneys contribute to HFD-induced hypertension. The first are data from spontaneously hypertensive rat (SHR), a commonly used hypertension rat model. High-fat diet causes hypertension and coincides with increased intrarenal lipid concentrations, oxidative stress, renal inflammation, and activation of renal RAS [51]. Secondly, dysregulated sodium transport in the kidneys leads to hypertension [52]. High-fat diets have been found to induce hypertension accompanying by impairing several sodium transporters in the kidneys, like Na^+^/Cl^−^ cotransporter (NCC), sodium hydrogen exchanger type 3 (NHE3), and Na-K-2Cl cotransporter (NKCC2) [30,53,54]. Another line of evidence comes from the activation of the renal RAS in high-fat-diet-induced hypertension and kidney injury [30,55,56]. It is well known that the kidney is a principal target for the various components of the RAS that are implicated in hypertension and kidney disease [57]. Accordingly, it is suggested that the intrarenal RAS activation plays an important role in hypertension induced by high-fat diet.

Although these organ systems have shown their potential roles in high-fat-diet-induced hypertension, little is known regarding programming effects of maternal HFD in the brain, heart, kidneys, and vessels in hypertension of developmental origins.

## 4. Hypertension of Developmental Origins: The Impact of Maternal High-Fat Diet

### 4.1. Animal Models of Maternal HFD-Induced Offspring Hypertension

A growing number of animal models have been generated to study hypertension of developmental origins, as reviewed elsewhere [14,58]. Imbalanced maternal nutrition can program the fetus resulting in hypertension in later life. Table 1 summarizes animal studies documenting offspring hypertension in animal models of high-fat diet, restricting the exposure to critical periods during early development [21,59,60,61,62,63,64,65,66,67,68,69,70,71]. In this review, we only considered studies reporting offspring outcomes starting from childhood.

Rats are the most frequently used animals. Other species such as rabbits [21] and mice [66] have also been used to study hypertension of developmental origins programmed by maternal HFD. In view of the fact that each month of the adult life of a rat corresponds to 3 human years [72], Table 1 shows the timing of developing hypertension in rats ranging from 12 weeks to 1 year of age, which corresponds to humans from childhood to adulthood. In rodents, different maternal HFDs with fat fractions ranged from 18.7% to 58% energy as fat, which were close to previous studies [7,8]. Notably, maternal HFD-induced responses of offspring BP could be increased [21,59,60,61,62,63,64,65,66,67,68,69,70,71] but also unaltered [30,61], mainly depending on sex, age, species, and varied fatty acid compositions. In Table 1, most BP data were obtained from the tail cuff method, except some studies using direct arterial catheter [21] or telemetry method [61,62]. Though BP data detected from the tail cuff method have been reported to correlate well with findings of direct arterial catheter and telemetry methods [73], part of the increased BP in offspring may be related to an increase in sympathetic nerve activity [74].

Additionally, maternal HFD-induced offspring hypertension is associated with developmental programming in several organs, including the kidneys [63,64,67,68,69,70,71], vessels [60,61,62,65,66], and brain [21]. Notably, offspring hypertension does not essentially correspond to obesity in response to maternal HFD. Offspring obesity may or may not appear in these maternal HFD-induced hypertension models. What is absent in the literature is animal models used for studying maternal HFD-induced hypertension target organ damage. Although maternal HFD induced programming effects in various organs, the contributions of organ-specific programming on damage in target organs have not been yet extensively studied in the above-mentioned animal models.

Of note is that the development of hypertension may follow the two-hit model. Recent evidence suggests a “two-hit” hypothesis that illuminates the developmental programming of adult diseases [75]. Hypertension can develop with two sequential hits, the first hit being the response to a prenatal insult, followed by the second hit in response to ongoing programming induced by the first hit. Since the first hit alone may not be sufficient to alter the adult phenotype, another insult may act as a second hit to amplify the underlying defects culminating in a disease state. A number of two-hit models, hence, have been used to evaluate whether two distinct hits affect offspring outcomes synergistically or differently when combined as compared with either hit alone.

In some studies, maternal HFD was applied as the first hit. Maternal HFD causes morphological functional changes of fetal organs, which alone might not be sufficient to alter the adult phenotype. The second hit could amplify ongoing programming processes triggered by maternal HFD, culminating in a disease state. So far, some two-hit models have been used to evaluate whether two distinct hits affect offspring hypertension synergistically or differently when combined as compared with maternal HFD alone. For example, models of a maternal plus post-weaning HFD [68,71] and combined maternal HFD and bisphenol A exposure [69] have been established to study hypertension of developmental origins. To sum up, animal models with various maternal HFDs during different fetal developmental stages generate the same outcome―hypertension in adult life. These findings suggest there might be major basic mechanisms behind hypertension of developmental origins.

### 4.2. Proposed Mechanisms Underlying Maternal HFD-Induced Offspring Hypertension

**Figure 2:**
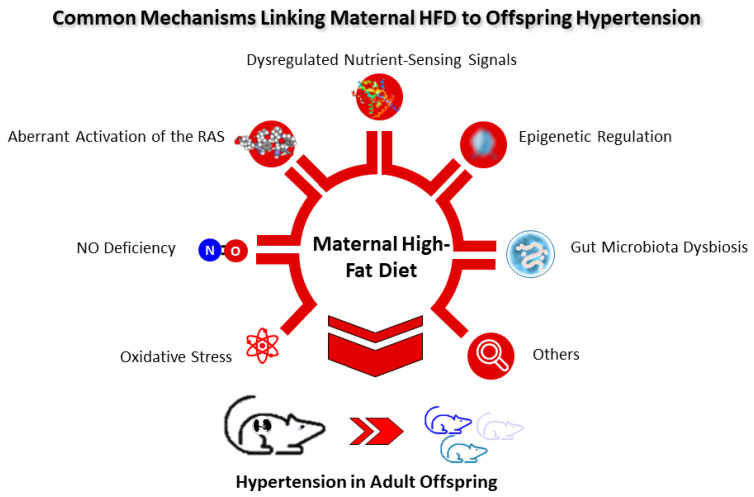
Schematic diagram of the common mechanisms linking maternal high-fat diet to offspring hypertension.

To date, several main mechanisms underlying hypertension of developmental origins have been proposed, such as oxidative stress, NO deficiency, aberrant activation of the RAS, dysregulated nutrient-sensing signals, epigenetic regulation, gut microbiota dysbiosis, etc. [14,57,58,76,77,78,79]. Among them, some are interconnected with maternal HFD and will be discussed in turn (Figure 2).

#### 4.2.1. Oxidative Stress

Oxidative stress, a disturbance in pro-oxidant/antioxidant balance, has been implicated in the pathophysiology of compromised pregnancy and adverse fetal outcomes [80]. During fetal development, the existence of excessive ROS under adverse intrauterine conditions prevails over the antioxidant defense system and causes fetal programming, leading to oxidative stress-related hypertension in adult offspring [81]. Prior reviews revealed that there are a number of maternal insult stimuli connected to oxidative stress in mediating offspring [14,81].

Several mechanisms accompanying by oxidative stress behind hypertension of developmental origins have been reported, including upregulation of ROS-producing enzymes [82], excessive ROS generation [69,83], increases in lipid peroxidation [84], elevated oxidative DNA damage [85], increased peroxynitrite formation [86], and decreased antioxidant capacity [63].

Table 1 shows that increased oxidative stress in the vessels [60,66] and kidneys [63,64,68,69] is associated with maternal HFD-induced hypertension in adult offspring. A commonly used marker of lipid peroxidation, malondialdehyde (MDA), has been used to detect oxidative damage and shown increased in the offspring kidneys in maternal HFD models [63,64]. Additionally, 8-hydroxydeoxyguanosine (8-OHdG), an oxidized nucleoside of DNA, is the most frequently detected DNA lesion [87], whose staining was significantly increased in the kidneys of adult rat offspring born to dams received HFD [68,69]. These findings provide the connections between maternal HFD and oxidative stress that underlie the hypertension of developmental origins.

#### 4.2.2. NO Deficiency

NO depletion in pregnancy can cause fetal programming, leading to programmed hypertension in adult offspring [76,88,89]. Prior work revealed that maternal NO deficiency alters a wide range of signaling pathways using the transcriptomic analysis [77]. Among them, several redox-sensitive signaling pathways contribute to the development of hypertension [90]. Additionally, NO deficiency in embryonic kidneys induced by ADMA can impair nephrogenesis and alter several genes related to hypertension of developmental origins [91]. Conversely, early interventions targeting the NO pathway could be used for reprogramming hypertension in different models of developmental programming [76,89].

As shown in Table 1, reduced NO in the vessels [60,65,66] and kidneys [63,68,69] is related to maternal HFD-induced offspring hypertension. Former work reveals that ADMA-related ROS–NO imbalance in early life causes hypertension in adult life [76]. In a combined maternal HFD and bisphenol A exposure [69], adult offspring develop hypertension coinciding with increased ADMA. However, maternal HFD alone increased offspring BP but has a negligible effect on ADMA level. A previous study reported that atorvastatin can reduce ADMA by increasing its metabolism to protect adult rats against high-fat diet-induced endothelial dysfunction [41]. As many currently used drugs have ADMA-lowering properties that can restore ADMA-NO balance [92], a deeper understanding of the reprogramming effects of NO-targeted intervention in HFD-induced programmed hypertension is warranted.

#### 4.2.3. Aberrant Activation of the RAS

The RAS, a major regulatory network of BP, is tightly connected with hypertension of developmental origins [57]. The RAS consists of several angiotensin (Ang) peptides that regulate BP through distinct receptors [93]. The classic RAS can be defined as the angiotensin-converting enzyme (ACE)/angiotensin (ANG) II/angiotensin II type 1 receptor (AT1R) axis. Activation of the classic RAS elicits vasoconstriction, oxidative stress, and inflammation, resulting in hypertension [94]. Maternal HFD-induced offspring hypertension is related to the aberrant activation of the classic RAS, represented by increases in increased renal mRNA expression of *Agt* and *Ace* and protein level of AT1R [67].

Conversely, the non-classic RAS, composed by the ACE2/Ang-(1–7)/Mas receptor, can counterbalance the adverse effects of ANG II [93]. Another study reported that renal Ang-(1–7) level was decreased in 16-week-old male offspring born to dams that received HDF [68]. Prior research indicated there is a transient biphasic response with downregulation of classic RAS axis in neonatal stage that becomes normalized with age [94]. Maternal HFD may disturb this normalization; hence, the classic RAS axis is aberrant activation, resulting in the rising BP in adult offspring.

Given the fact that only few studies addressed the impact of RAS in maternal HFD-induced hypertension, there is need for further investigation of this research gap. Considering maternal HFD increased offspring’s BP coinciding with aberrant activation of the classic RAS, more work is required to explore whether blockade of the RAS can be used as reprogramming interventions for maternal HFD-induced programmed hypertension.

#### 4.2.4. Dysregulated Nutrient-Sensing Signals

During gestation, nutrient-sensing signals regulate fetal metabolism in response to maternal nutritional status [95]. Different signaling pathways that detect intracellular and extracellular levels of specific nutrients, such as fats, are coordinated at the organismal level via hormonal signals [96]. Accordingly, maternal nutritional imbalance resulting in dysregulation of nutrient-sensing signals cause a crucial impact on hypertension of developmental origins [14,93].

Maternal HFD-induced offspring hypertension is correlated to inhibitory AMP-activated protein kinase (AMPK)/peroxisome proliferator-activated receptor-γ (PPARγ) coactivator-1α (PGC-1α) pathway in offspring kidneys [68]. AMPK can phosphorylate PGC-1α. PGC-1α binds to PPARγ and coactivates PPARγ to facilitate the expression of specific sets of PPAR target genes participating in hypertension [97]. A growing body of evidence indicates that downregulation of nutrient-sensing signals, such as AMPK and PGC-1α, is related to hypertension of developmental origins, while AMPK activation can serve as a reprogramming strategy to protect offspring against adverse programmed processes [98]. Although a potential link between nutrient-sensing signals and maternal HFD underlying hypertension of developmental origins exists, whether these signals impact maternal HFD-induced adverse offspring outcomes in an organ-specific manner remains unclear.

#### 4.2.5. Epigenetic Regulation

During pregnancy, epigenetic mechanisms are involved in programming gene expression for fetal development [99]. Recent studies suggest that DNA methylation, histone modification, and noncoding RNAs may be one of the epigenetic mechanisms that program the effects of early-life habits on later-life risk of adult diseases, including hypertension [78,100].

Using next-generation RNA sequencing (NGS) analysis, our prior work reported maternal HFD significantly altered renal transcriptome in 1-week-old female offspring [30]. In total, 251 differential expressed genes (DEGs) (154 up- and 97 downregulated genes) were identified. Among them, several genes were related to regulation of BP, such as *Agtr1b* and *Ace* belonging to the RAS, *Ddah1* in the NO signaling pathway, and sodium transporter *Slc12a3*. Notably, a maternal HFD also induces differential alterations of gene expression in the placenta [100], brain [101], and heart [102] in offspring. These data demonstrate that epigenetic regulation may participate the developmental programming of adult diseases in an organ-specific manner.

As well, maternal HFD leads to offspring hypertension and was relevant to increased leptin promoter hypomethylation and leptin expression in adipose tissues of HFD-exposed rat offspring [59]. Similarly, another study demonstrated that maternal HFD may program sympatho-excitatory capacity to induce hypertension in adult rabbit offspring attributed to increased leptin receptor [58]. Hypothalamic leptin signaling can activate specific melanocortin receptors located on sympathetic neurons and consequently result in sympathetic activation of the heart and kidneys and, finally, elevated BP [103]. Thus, maternal HFD can regulated certain genes involved in the regulation of BP. However, the underlying epigenetic mechanisms await further clarification.

#### 4.2.6. Gut Microbiota Dysbiosis

Within the gut reside various microbes coexisting with the host in a mutually beneficial relationship, and thus gut microbiota has potential to affect human health and disease [104]. During pregnancy and lactation, the mothers share gut microbiota and derived metabolites with their offspring, which illuminate the impact of maternal influences in the development of offspring’s gut microbiota [105].

A meta-analysis including 15 studies indicated that intake of high saturated fat may negatively affect microbiota richness and diversity [106]. Maternal HFD was reported to reduce α-diversity in offspring’ microbiota [107]. Loss of α-diversity appears as the most constant finding of gut microbiota dysbiosis, leading to many human diseases [108]. Additionally, a maternal HFD programs offspring’s hypertension coincides with an increased *Firmicutes* to *Bacteroidetes* (F/B) ratio. This was tied well with hypertension models showing the F/B ratio was increased and served as a microbial marker of hypertension [109]. Additionally, reduction of beneficial microbes was also a hallmark of gut microbiota dysbiosis [104]. The abundance of both beneficial bacterial strains *Lactobacillus* and *Akkermansia* [110,111], were reduced in the maternal HFD model [67,71].

Moreover, maternal HFD increased offspring’s BP accompanying by alterations of gut microbiota-derived metabolites [67,71]. Microbial metabolites such as short-chain fatty acids (SCFAs), trimethylamine (TMA), and trimethylamine N-oxide (TMAO) are involved in BP regulation [112,113,114]. Maternal HFD was reported to reduce fecal propionate level in 3-week-old offspring [71]. As propionate has vasodilatory action via mediating SCFA receptors [112], this finding suggests reduced SCFA might be a possible reason contributing to maternal HFD-induced hypertension. Maternal HFD also caused the increases of TMA levels and decreases of TMAO-to-TMA ratio in adult rat offspring [71]. As microbiota-derived metabolites TMA and TMAO are risk factors for cardiovascular disease [113,114], whether HFD-induced programmed hypertension can be prevented by reducing accumulation of TMA and TMAO warrants further investigation.

#### 4.2.7. Others

With regard to the multiple negative aspects of maternal HFD on offspring outcomes, other possible mechanisms might be involved, for example, dysregulation of H_2_S or sex differences. Hydrogen sulfide (H_2_S), the third gasotransmitter, has emerged as an important regulator of BP [115]. Increasing evidence supports the use of H_2_S-based interventions as a reprogramming approach to protect offspring against hypertension of developmental origins [116]. One previous study revealed maternal HFD caused decreases of plasma H_2_S levels and renal H_2_S-releasing activity in male rat offspring [70]. These findings suggest that a link between HFD and H_2_S might be behind hypertension of developmental origins, although this remains speculative. Sex-dependent differences exist in hypertension of developmental origins [117,118]. It has been noted that male offspring are more susceptible to be hypertension than female offspring [117,118]. This difference has led many researchers to work on predominately male animal research, as listed in Table 1. However, one study reported that maternal HFD programs hypertension in female but not male offspring [61]. Another study revealed there is no difference of maternal HFD on the development of hypertension in each sex [30]. More research on sex differences is required as they may become a potential mechanism in hypertension programmed by maternal HFD.

## 5. Reprogramming Strategies

With a deeper understanding of the mechanisms underlying maternal HFD-programmed hypertension, the development of mechanism-targeted strategies holds potential for reprogramming. So far, early-life interventions to offset mechanisms governing hypertension of developmental origins that have been evaluated range from avoidance of risk factors, nutritional interventions, pharmacological therapies, to lifestyle modification [14,58,81].

**Figure 3:**
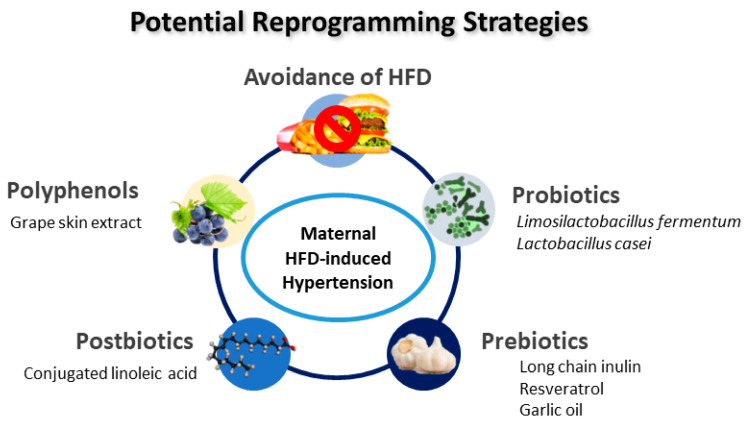
Schematic diagram of the potential reprogramming strategies for prevention of maternal high-fat-diet-induced offspring hypertension.

Animal models have been essential in providing potential reprogramming strategies. As described in Table 1, several interventions have been used as reprogramming strategies to prevent hypertension in offspring exposed to maternal HFD, including grape skin extract [60], *Limosilactobacillus fermentum* [64], conjugated linoleic acid [65], long chain inulin [67], *Lactobacillus casei* [67], resveratrol [68,69], and garlic oil [70]. A schematic summarizing the potential reprogramming interventions for maternal HFD-induced hypertension of developmental origins is presented in Figure 3.

Interestingly, most reprogramming interventions used in maternal HFD models are targeted on gut microbiota. Probiotics and prebiotics have long been known for their benefits in human health [119,120], including treating hypertension [121], while less attention has been paid to their preventive aspect on hypertension of developmental origins [58]. Probiotic treatment with *Limosilactobacillus fermentum* [64] or *Lactobacillus casei* [67] in pregnancy and lactation prevents the development of hypertension in adult offspring exposed to maternal HFD. Additionally, long-chain inulin, a prebiotic, causes a beneficial protective effect against maternal HFD-induced offspring hypertension [67]. Similarly, resveratrol could be used to reprogram maternal HFD-induced hypertension due to its probiotic properties [68,69]. Another study showed that garlic oil, one of the prebiotic foods, protected maternal HFD-induced hypertension in adult rat progeny [70]. Its beneficial effects include increased α-diversity, increased plasma SCFA levels, and increased proportions of beneficial bacteria *Bifidobacterium* and *Lactobacillus*.

Apart from probiotics and prebiotics, postbiotics are another gut microbiota-based modality [122]. Postbiotics are bioactive compounds made from metabolic by-products of live probiotic bacteria [122]. Conjugated linoleic acid is a gut microbiota-derived metabolite from dietary polyunsaturated fatty acids. As a postbiotic, maternal conjugated linoleic acid supplementation reversed maternal HFD-induced offspring hypertension [65].

Furthermore, former work reveals that specific nutrient intake can be advantageous for protecting offspring from hypertension of developmental origins in various animal models [123,124]. One of them is polyphenol [124]. One previous study showed that supplementation with grape skin polyphenols during gestation and lactation protects against hypertension induced by a maternal HFD [60].

To summarize, current evidence from animal models supports that early-life interventions such as gut microbiota-targeted therapies may be able to prevent the development of hypertension programmed by maternal HFD in a desired favorable direction.

## 6. Conclusions and Perspectives

Previous research has shown that maternal HFD causes a variety of adverse offspring outcomes later in life. In this review, we outlined recent advances supporting the influence of maternal HFD on offspring hypertension. Reflecting current knowledge of animal models, our review also shed light on prevention of maternal HFD-induced hypertension via innovative reprogramming strategies. Unfortunately, animal models of maternal HFD are not without limitations. There is no one-size-fits-all model to induce the same adverse offspring outcomes (e.g., hypertension or obesity) in response to maternal HFD. Part of the issue is limited not simply to practical issues regarding study design (compositions of fats, duration, and age at measure) but also to the confounding effects of genetic variation (sex, animal species, and background). Future work could point to improve comparability across studies using “identical” maternal HFD models.

To move the field forward, some unsolved aspects toward clinical translation need to be considered. Although several core mechanisms were addressed in the current review, we may not completely understand the full picture of mechanisms behind maternal HFD-induced offspring hypertension. Considering maternal HFD can program various organs resulting different phenotypes in adult offspring, it is imperative that additional studies simultaneously evaluate organ-specific programming effects in an experiment. What is absent from the literature is a deeper understanding of whether maternal HFD can program offspring hypertension leading to damage of target organs, such as the brain, heart, and kidneys.

Studies to date have found that gut microbiota-targeted therapies can reprogram maternal HFD-induced hypertension. Nevertheless, none of them have been translated in human studies. Despite results from human studies revealing probiotics and prebiotics in the treatment of maternal conditions during pregnancy being beneficial, currently their effectiveness in protecting offspring against hypertension remain largely unclear. Future work in large trials is required to better identify appropriate probiotic species and prebiotics for pregnant women consuming HFD for preventing hypertension in their children.

In summary, the growing body of knowledge suggests maternal dietary habits with excessive fat intake during gestation and lactation should be avoided to minimize offspring risk for developing hypertension in later life. After a greater understanding of maternal HFD-induced offspring hypertension and a remarkable growth achieved in reprogramming strategies, we believe that translating animal results into clinical application is a valuable approach that could curtail the global pandemic of hypertension.

## Figures and Tables

**Table 1:** Programming effects of maternal high-fat diet on offspring hypertension.

Energy Percent from Fat in Maternal High-Fat Diet	Species/Sex	Intervention Period	Offspring Obesity	Programming Effects	Age at Measure	Ref.
13.4%	Rabbit/M+F	Pregnancy and Lactation	No	Increased central leptin signaling and sympathetic responsivity	20 weeks	[21]
18.7%	SD rat/M	Pregnancy	No	Increased leptin expression and leptin promoter hypomethylation in adipose tissues	1 year	[59]
24%	Wistar rat/M	Lactation	Yes	Decreased plasma and mesenteric arteries antioxidant activities, and decreased NO	22 weeks	[60]
25.7%	SD rat/M+F	Lactation	Yes in females	Endothelial dysfunction	25 weeks	[61]
25.7%	SD rat/F	Pregnancy and Lactation	Yes	Endothelial dysfunction	180 days	[62]
31%	Wistar rat/M+F	Pregnancy and Lactation	ND	Reduced SOD activity and increased lipid peroxidation in the kidneys	90 days	[63]
31%	Wistar rat/M	Pregnancy and Lactation	ND	Increased oxidative stress in the kidneys	100 days	[64]
45%	SD rat/M	Pregnancy and Lactation	Yes	Endothelial dysfunction and reduced NO	130 days	[65]
45%	C57BL6J mice/M	Pregnancy and Lactation	Yes	Endothelial dysfunction, increased ROS, and reduced NO in femoral artery	30 weeks	[66]
58%	SD rat/M	Pregnancy and Lactation	No	Increased renal AT1R expression and shifts in gut microbiota	16 weeks	[67]
58%	SD rat/M	Pregnancy and Lactation	No	Increased renal oxidative stress, decreased urinary NO level, and decreased renal Ang-(1–7) level	16 weeks	[68]
58%	SD rat/M	Pregnancy and Lactation	No	Increased renal oxidative stress and decreased urinary NO level	16 weeks	[69]
58%	SD rat/M	Pregnancy and Lactation	Yes	Dysregulated H_2_S-generating pathway and shifts in gut microbiota	16 weeks	[70]
58%	SD rat/M	Pregnancy and Lactation	No	Dysregulated nutrient-sensing signals and shifts in gut microbiota	16 weeks	[71]

Studies tabulated according to energy percent from fat in maternal diet, species, and age at measure. SD = Sprague Dawley; M = male; F = female; ND = Not determined; NO = nitric oxide; ROS = reactive oxygen species; SOD = superoxide dismutase; AT1R = angiotensin II type 1 receptor; H_2_S = hydrogen sulfide.

## Data Availability

All data are contained within the article.
